# Protective Effect of Chlorogenic Acid on Human Sperm: In Vitro Studies and Frozen–Thawed Protocol

**DOI:** 10.3390/antiox10050744

**Published:** 2021-05-07

**Authors:** Daria Noto, Giulia Collodel, Daniela Cerretani, Cinzia Signorini, Laura Gambera, Andrea Menchiari, Elena Moretti

**Affiliations:** 1 Department of Molecular and Developmental Medicine, University of Siena, 53100 Siena, Italy; noto@student.unisi.it (D.N.); giulia.collodel@unisi.it (G.C.); cinzia.signorini@unisi.it (C.S.); 2 Department of Medicine, Surgery and Neurosciences, University of Siena, 53100 Siena, Italy; daniela.cerretani@unisi.it; 3 Fertility Center, AGI Medica, 53100 Siena, Italy; lauragambera@agimedica.it; 4 Department of Business and Law, University of Siena, 53100 Siena, Italy; andrea.menchiari@unisi.it

**Keywords:** AMPK, chlorogenic acid, cryopreservation, DNA integrity, human sperm, isoprostanes, MDA, membrane mitochondrial potential, oxidative stress, TEM

## Abstract

The study evaluated the chlorogenic acid (CGA) antioxidant potential on oxidative stress (OS) induced in vitro in human spermatozoa and during cryopreservation procedure. Swim-up selected spermatozoa were treated with 100 µM CGA, 100 µM H_2_O_2_ to induce lipid peroxidation (LPO), and with both compounds and the effects on mitochondrial membrane potential (MMP) by JC-1, DNA integrity by acridine orange (AO), and sperm ultrastructure by transmission electron microscopy (TEM), were evaluated. CGA antioxidant activity was assessed by measuring malondialdehyde (MDA) and F_2_-isoprostanes (F_2_-IsoPs) in the media. The CGA protective activity and the immunolocalization of Phospho-AMPKα (Thr172) were explored in frozen-thawed sperm. CGA was not toxic for sperm motility, DNA integrity and MMP. The increase in MDA (*p* < 0.05) and F_2_-IsoPs (*p* < 0.001), DNA damage (*p* < 0.01) and low MMP (*p* < 0.01) levels after H_2_O_2_ treatment were reduced in presence of CGA as well as the percentage of broken plasma membranes (*p* < 0.01) and altered acrosomes (*p* < 0.01) detected by TEM. Treated frozen-thawed spermatozoa showed increased sperm motility (*p* < 0.01), DNA integrity (*p* < 0.01), MMP (*p* < 0.01), reduced MDA (*p* < 0.01) and increased sperm percentage with Phospho-AMPKα labelling in the head (*p* < 0.001). CGA can be used to supplement culture media during semen handling and cryopreservation where OS is exacerbated.

## 1. Introduction

Assisted reproductive techniques (ARTs) represent an important resource in the treatment of infertility. However, gamete handling procedures such as cryopreservation, centrifugation, light exposure, pH variations and temperature [1,2,3] make cells mostly vulnerable to oxidative stress (OS) [4]. In particular, cryopreservation represents an important resource in the infertility treatment especially in patients undergoing oncological therapies, in men with severe spermatogenic dysfunction [5] and in human sperm cryobanking. On the other hand, freezing process induces reactive oxygen species (ROS) formation, DNA fragmentation, apoptosis and lipid peroxidation (LPO) [6] and damages crucial genes involved in fertilization and early embryo development [7].

Generally, a physiological amount of ROS is essential for sperm motility, capacitation, acrosomal reaction and sperm-oocyte interaction [8]. However, ROS overproduction causes different damages to the spermatozoa at membrane, DNA, and protein levels [9]. In particular, sperm membranes are extremely sensitive to OS due to the high content of polyunsaturated fatty acids (PUFAs), resulting in LPO [8]. Products of this oxidative event are aldehydes, such as malondialdehyde (MDA) and 4-hydroxynonenals (4-HNE), conjugated diene compounds and isoprostanoids and can be measured as OS markers [10,11]. MDA levels are a highly sensitive and specific measurement of LPO in semen [12]. Recently, F_2_-isoprostanes (F_2_-IsoPs) are also considered a good available marker of LPO and can be used to evaluate the oxidative status in many human diseases even in male infertility; F_2_-IsoPs have been reported to be detectable both in sperm membrane and seminal plasma [13,14]. F_2_-IsoPs, products of arachidonic acid oxidation, are initially formed in situ on phospholipids and then released in free form in biological fluids.

A strategy to overcome the problems related to OS resulting from gamete handling procedures could be the supplementation of media with antioxidants during sperm manipulations [15,16]. To this end, many studies have reported the scavenging ability of antioxidant compounds against OS induced in vitro and in freezing procedures both in human [17,18,19,20] and animal [21,22,23,24,25,26] spermatozoa. Recently, Alamo et al. [27] showed that resveratrol reduces the detrimental effects of benzo-α-pyrene on motility, chromatin integrity, membrane, and mitochondria of human sperm.

Chlorogenic acid (CGA), an ester derived from caffeic acid and quinic acid, is one of the phenolic acid compounds present in several foods such as vegetables, fruits, coffee, and tea [28]. It displays different pharmacological activities such as antioxidant, anti-inflammatory, anti-microbial, hepatoprotective, anticancer, anti-lipidemic, and anti-diabetic [29]. A concentration of 100 µM CGA yielded beneficial effects on sperm motility, viability, and plasma membrane integrity in boar semen during freezing [30] and cooling at 15 °C [31,32]. CGA also displayed a positive effect in in vitro production of porcine embryos and a protection against DNA damage induced by OS in oocytes [33]. Furthermore, it was reported that CGA increases the phosphorylation of AMPK [29], protein involved in the regulation of sperm motility, preservation of sperm membrane integrity and mitochondrial membrane potential (MMP) [34].

The working hypothesis was first to test the effect of CGA on OS induced in vitro in human sperm by assessing sperm motility, MMP, DNA integrity and antioxidant ability. Then, we supplemented the freezing medium with CGA during sperm cryopreservation and assessed the potential protective effect of this compound after thawing. Phospho-AMPKα (Thr172) immunofluorescence labelling was carried out on frozen-thawed samples in order to evaluate the possible differences between treated and untreated samples.

## 2. Materials and Methods

### 2.1. Study Design

**Scheme 1:**
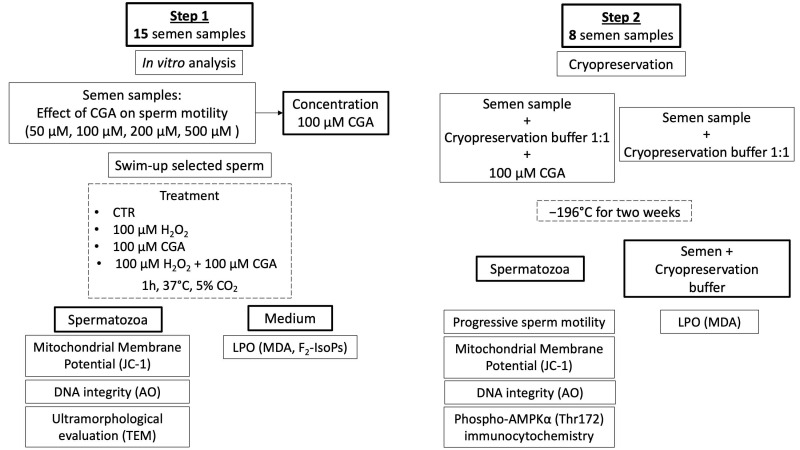
Flow chart representing the study design of the research.

The study comprised two different steps (Scheme 1):

Step 1: an analysis of the protective action of CGA on OS induced in vitro with H_2_O_2_.
Step 2: an applicative study of the effect of CGA during semen cryopreservation.

### 2.2. Semen Samples

Semen samples from 15 normozoospermic donors (aged 24–30 years) attending the Department of Molecular and Developmental Medicine were used for in vitro studies (step 1).

Semen samples of 8 men (aged 28–37 years) attending AGI Medica, Fertility Center lab for semen analysis (Siena, Italy) were used for cryopreservation experiments (step 2).

Participants signed an informed written consent before the participation in this research, declaring their acceptance that their semen samples might be used for scientific purposes.

Semen samples were collected by masturbation after 3–5 days of sexual abstinence and analysed after liquefaction for 30 min at 37 °C. Semen volume, pH, sperm concentration, and motility were evaluated according to the World Health Organization guidelines [35].

### 2.3. Step 1: In Vitro Studies

#### 2.3.1. Evaluation of CGA Effect on Sperm Motility

CGA was purchased from Sigma-Aldrich (St. Louis, MO, USA); a stock solution 1 mM was prepared by dissolving the powder in distilled water and stored at 4 °C. Semen samples were incubated at 37 °C and 5% CO_2_ for 1 h with different concentrations of CGA (50 μM, 100 μM, 200 μM, 500 μM). Sperm motility was evaluated using a Burker counting chamber identifying progressive and non-progressive motility and immotile sperm [35]. Sperm treated in the same conditions but without CGA were used as controls. The experiment was carried out in 10 samples.

#### 2.3.2. Swim-Up to Select Motile Sperm

In order to select a motile sperm population, the swim-up technique was performed. Briefly, 0.5 mL of Sperm Washing Medium IrvineScientific^®^ (Santa Ana, CA, USA) was gently stratified above 0.5 mL of each semen sample in sterile conical centrifuge tubes. The tubes were inclined at 45° angle and incubated for 45 min at 37 °C and 5% CO_2_. Afterward, 0.5 mL of the uppermost medium, containing motile sperm, was collected, divided into 4 aliquots, and treated as follows:

- Untreated sperm as control;
- Sperm treated with 100 µM H_2_O_2_;
- Sperm treated with 100 µM CGA;
- Sperm treated with both 100 µM H_2_O_2_ and 100 µM CGA.

All the aliquots were incubated at 37 °C and 5% CO_2_ for 1 h. After incubation, the aliquots were centrifuged at 400× *g* for 15 min. The recovered supernatant was immediately examined under a microscope ensuring the absence of cells and then stored at −80 °C until MDA and F_2_-IsoP levels were evaluated. Spermatozoa were used for JC-1 and AO determinations.

#### 2.3.3. JC-1 to Evaluate Mitochondrial Membrane Potential (MMP)

Alterations in MMP of spermatozoa from upper swim-up fraction treated with 100 µM CGA, 100 µM H_2_O_2_ and with both 100 µM H_2_O_2_ and 100 µM CGA were evaluated using the cation 5,5′,6,6′-tetrachloro-1,1′,3,3′-tetraethylbenzimidazolcarbocyanine iodide (JC-1) dye (Molecular Probes, Eugene, OR, USA). In brief, a final sperm concentration of 2 × 10^6^/mL were dark incubated with 1 μg/mL JC-1 dye (diluted in PBS) for 20 min at 37 °C. Observations were evaluated with a Leitz Aristoplan fluorescence Microscope (Leica, Wetzlar, Germany) equipped with a 490 nm excitation light and 530 nm barrier filter. At least 300 sperm were scored at 1000× magnification. Sperm with high MMP showed a red fluorescence at midpiece level due to the presence of JC-1 aggregates, those with low MMP a diffuse green signal since JC-1 remains in monomeric form. The experiment was carried out in 10 samples. Results are reported as a percentage of sperm with high MMP.

#### 2.3.4. Acridine Orange (AO) to Evaluate DNA Integrity

The DNA integrity of spermatozoa from upper swim-up fraction treated with 100 µM CGA, 100 µM H_2_O_2_ and with both 100 µM H_2_O_2_ and 100 µM CGA was assessed by acridine orange (AO) test as reported in Moretti et al. [36].

The slides were observed and evaluated with a Leitz Aristoplan fluorescence Microscope (Leica, Wetzlar, Germany) equipped with a 490 nm excitation light and 530 nm barrier filter. At least 300 sperm were scored at 1000× magnification. Spermatozoa with double stranded DNA (dsDNA) displayed a green fluorescence. Sperm with denatured DNA showed a spectrum of yellow orange to red fluorescence. The experiment was carried out in 10 samples. The results were expressed as percentage of sperm with dsDNA (green fluorescence).

#### 2.3.5. Malondialdehyde (MDA) Level Assessment

The swim-up media, stored at −80 °C, were thawed to evaluate the extent of LPO induced with 100 μM H_2_O_2_ and the potential scavenging activity of 100 μM CGA by measuring free MDA levels according to Shara et al. [37]. Briefly, 500 μL of each sample was added to 500 μL of 0.04 M Tris (hydroxymethyl) methylamine (pH 7.4) and 0.01% butyl hydroxytoluene in acetonitrile (1:1 *v*/*v*) to avoid the artificial oxidation of polyunsaturated free fatty acids during the assay. The samples were centrifuged at 3000× *g* at 4 °C for 15 min. The swim-up media was used for MDA analysis after precolumn derivatization with 2,4-dinitrophenylhydrazine. The MDA-hydrazone was quantified by isocratic reversed-phase HPLC (Waters 600 E System Controller HPLC equipped with a Waters Dual λ 2487 detector, Milford, MA, USA) with UV detection at 307 nm. Each sample was assessed in duplicate, and the results are expressed in nmol of MDA per mL of swim-up medium. The experiment was carried out in 10 samples.

#### 2.3.6. F_2_-isoprostane Determination

The levels of free F_2_-IsoPs were measured in the media of upper swim-up fraction, stored at −80 °C, after treatment with 100 μM H_2_O_2_ and 100 μM CGA plus 100 μM H_2_O_2_ and untreated.

For the analysis, each sample was spiked with tetradeuterated derivative of PGF_2α_ (PGF_2α_-d_4_) (500 pg), as an internal standard. Then, each sample was applied to an octadecylsilane (C18) cartridge followed by an aminopropyl (NH_2_) cartridge and isoprostanes were eluted. After that, the F_2_-IsoP carboxylic group was derivatized as the pentafluorobenzyl ester whereas the hydroxyl groups were converted to trimethylsilyl ethers. Finally, F_2_-IsoP determinations were carried out by gas chromatography/negative ion chemical ionization tandem mass spectrometry (GC/NICI-MS/MS) analysis. The measured ions were *m/z* 299 and *m/z* 303 derived from the (M-181)^−^ precursor ions (*m/z* 569 and *m/z* 573) produced from the derivatized 15-F_2_t-IsoP (i.e., 8-iso-PGF_2α_, the most represented isomer for F_2_-IsoP measurement) and PGF_2α_-d_4_, respectively [38]. The experiment was carried out in 10 samples and the results expressed as pg/mL.

#### 2.3.7. Transmission Electron Microscopy (TEM)

Swim-up selected spermatozoa treated with 100 μM H_2_O_2_ and 100 μM CGA plus 100 μM H_2_O_2_ were processed for TEM. Samples were fixed in Karnovsky fixative at 4 °C for 2 h and washed in 0.1 M cacodylate buffer (pH 7.2) for 12 h. Then, spermatozoa were postfixed in 1% buffered osmium tetroxide for 1 h at 4 °C, dehydrated in a graded ethanol series (50%, 75%, 95%, 100%) and embedded in Epon Araldite. Ultrathin sections were obtained with the Supernova Ultramicrotome (Reichert Jung, Vienna, Austria), mounted on copper grids and stained with uranyl acetate and lead citrate. Stained grids were observed with a Philips CM12 TEM (Philips Scientific, Eindhoven, the Netherlands; Centro di Microscopie Elettroniche “Laura Bonzi”, ICCOM, Consiglio Nazionale delle Ricerche (CNR), Via Madonna del Piano, 10, Firenze, Italy).

The experiments were performed on 3 samples and assessed twice by two different examiners. For each sample, at least 300 sperm sections were evaluated and the abnormalities of acrosome, chromatin, axoneme, and the plasma membrane were quantified.

### 2.4. Step 2: Applicative Study

#### 2.4.1. Cryopreservation

Eight sperm samples were washed with Sperm Washing Medium IrvineScientific^®^ (Santa Ana, CA, USA), centrifuged at 400× *g* for 10 min, and excess supernatant removed. Two aliquots of each sample were obtained and freezing medium (TYB with Glycerol and Gentamicin; Irvine Scientific, Santa Ana, CA, USA) was added 1:1 (*v*:*v*) dropwise to the specimens and gently mixed. In the treated sample, the freezing medium was supplemented with 100 μM CGA to test its potential protective activity.

Then, samples were placed into paillettes with a final volume of 0.3 mL, located 30 min at 4 °C and finally dipped in liquid nitrogen at −196 °C.

Two weeks later, the paillettes were placed at 37 °C for 10 min and sperm motility was assessed.

Furthermore, the aliquots were centrifuged at 400× *g* and the supernatant, composed of medium without spermatozoa, was used for MDA levels evaluation; each sample was assessed in duplicate and the results expressed in nmol of MDA per mL of medium. Spermatozoa were used for MMP and DNA integrity determinations. The experiments were assessed twice by two different examiners.

#### 2.4.2. Immunocytochemistry

The immunolocalization of the phosphorylated form of AMPK was performed in cryopreserved spermatozoa treated with 100 µM CGA and untreated. Spermatozoa were washed in phosphate buffer saline (PBS), smeared on glass slides, air dried and fixed in 4% paraformaldehyde (PFA) for 15 min. After a treatment with blocking solution (PBS-BSA 1% NGS 5%) for 20 min, slides were incubated overnight at 4 °C with a primary antibody anti-Phospho-AMPKα (Thr172) (Cell Signaling Technology, Danvers, MA, USA) diluted 1:100. The reaction was revealed by an anti-rabbit antibody raised in a goat Alexa Fluor^®^ 488 conjugate (Invitrogen, Thermo Fisher Scientific, Carlsbad, CA, USA), diluted at 1:100. Incubation without the primary antibodies was used as control. Nuclei were stained with 4,6-diamidino-2-phenylindole (DAPI) solution (Vysis, Downers Grove, IL, USA). Observations were made with a Leica DMI 6000 Fluorescence Microscope (Leica Microsystems, Germany), and the images were acquired by the Leica AF6500 Integrated System for Imaging and Analysis (Leica Microsystems, Germany). The experiments were performed on 8 samples and assessed twice by two different examiners.

### 2.5. Statistical Analysis

Statistical analysis was performed with the SPSS version 17.0 for Windows software package (SPSS Inc, Chicago, IL, USA). The Kolmogorov-Smirnov test was used to verify the normality in the distribution of the variables. The Kruskal-Wallis test was used to compare the difference among different groups and then Dunnet Post Hoc test was applied to determine which groups differed statistically from each other. The Mann-Whitney test was applied to compare the differences between TEM variables (altered acrosome %, altered chromatin %, altered axoneme %, broken plasma membrane % in non-frozen samples) measured in two groups (sperm incubated with H_2_O_2_ and sperm with H_2_O_2_ + CGA) and MDA, AO, JC-1 in frozen samples treated with CGA and untreated. The Mann-Whitney U test was also used to compare the percentage of sperm with Phospho-AMPKα labelling revealed in frozen-thawed sperm treated with CGA and untreated. Data were reported as median (interquartile range, QR). *p* < 0.05 was considered significant.

## 3. Results

### 3.1. Effect of Chlorogenic Acid on Sperm Progressive Motility

Results showed that CGA did not have a statistically significant effect on sperm progressive motility at concentrations of 50 μM, 200 μM and 500 μM compared to control (CTR). The percentage of progressive sperm motility was increased when CGA was used at 100 µM respect to CTR (*p* < 0.05) (Table 1).

### 3.2. Effect of Chlorogenic Acid on Sperm DNA Integrity

**Figure 1:**
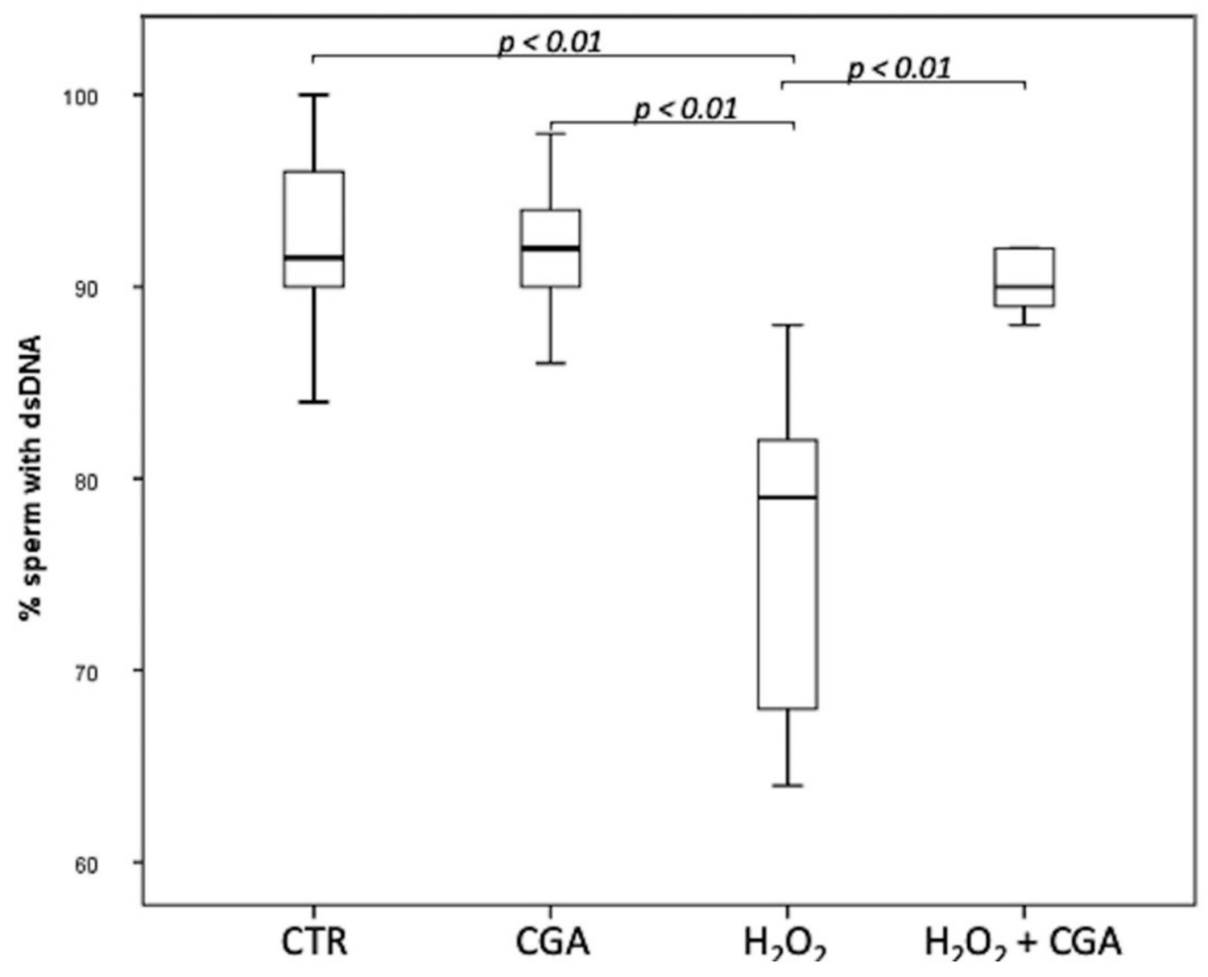
Median (IQR) of dsDNA percentage of swim-up selected sperm treated as follows: control (CTR), 100 µM chlorogenic acid (CGA), 100 µM H_2_O_2_ (H_2_O_2_), 100 µM H_2_O_2_ + 100 µM CGA (H_2_O_2_ + CGA). The experiment was repeated 10 times.

The effect of CGA on DNA integrity of swim-up selected sperm treated with 100 µM CGA, 100 µM H_2_O_2_ and with both 100 µM H_2_O_2_ and 100 µM CGA was assessed. Results showed that the percentage of the sperm with dsDNA significantly decreased in spermatozoa treated with 100 µM H_2_O_2_ (79.00; IQR: 67.50–83.50%) compared with CTR (91.50; IQR: 88.75–96.00%, *p* < 0.01) and with 100 µM CGA (92.00; IQR: 89.50–95.00%, *p* < 0.01). The concentration of 100 µM CGA did not show any effect on DNA integrity if used alone. Moreover, the percentage sperm with dsDNA was significantly higher in spermatozoa treated with 100 µM H_2_O_2_ plus 100 µM CGA (90.0; IQR: 88.75–92.00%) that those treated with 100 µM H_2_O_2_ (*p* < 0.01) (Figure 1).

### 3.3. Effect of Chlorogenic Acid on Mitochondrial Membrane Potential (MMP)

**Figure 2:**
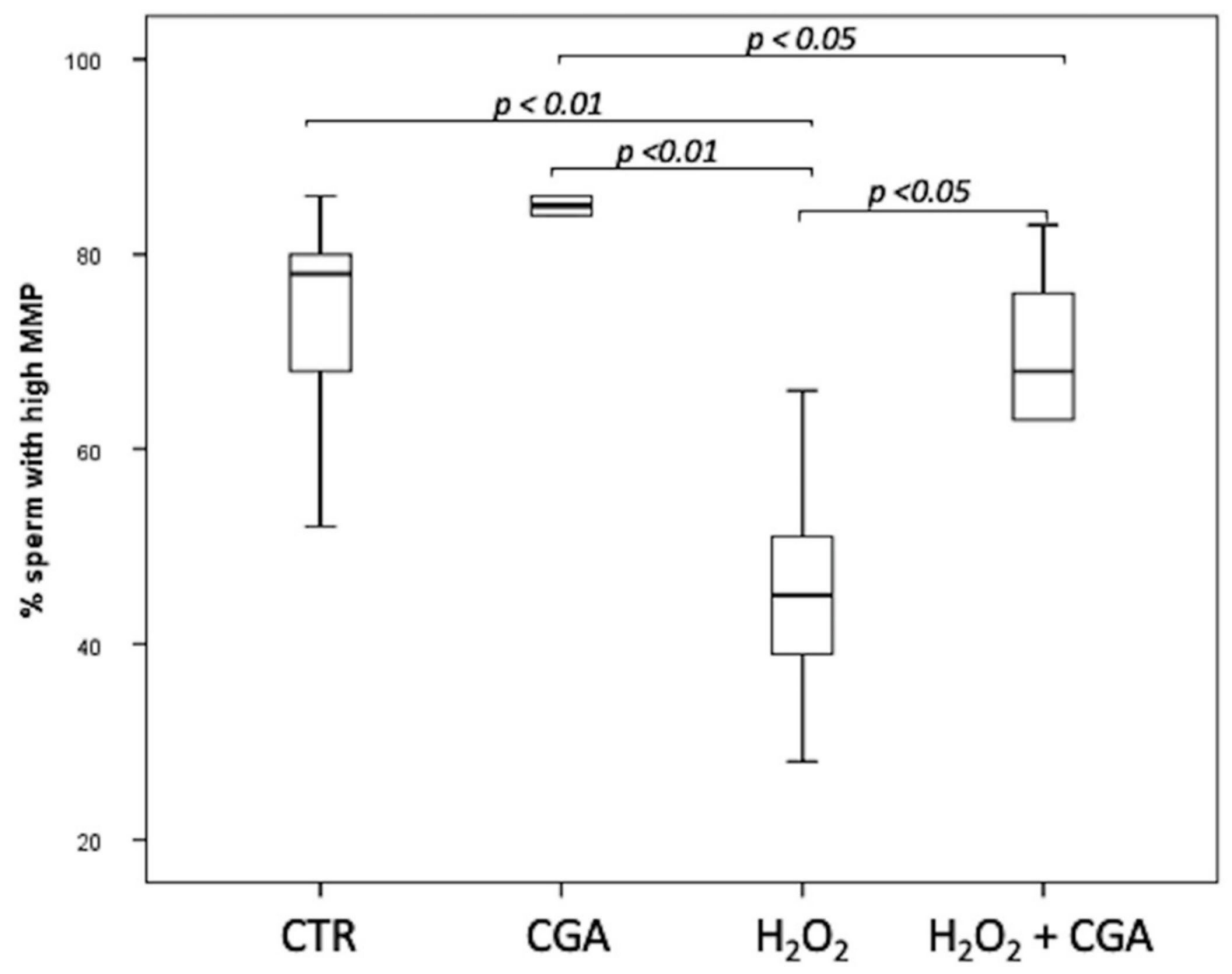
Median (IQR) of high MMP percentage of swim-up selected sperm treated as follows: control (CTR), 100 µM chlorogenic acid (CGA), 100 µM H_2_O_2_ (H_2_O_2_)_,_ 100 µM H_2_O_2_ + 100 µM CGA (H_2_O_2_ + CGA). The experiment was repeated 10 times.

The MMP of swim-up selected sperm treated with 100 µM CGA, 100 µM H_2_O_2_ and with both 100 µM H_2_O_2_ and 100 µM CGA was assessed. Results showed that the percentage of sperm with high MMP increased, although not significantly, in the samples treated with CGA alone (85.50; IQR: 79.75–87.00%) than those observed in CTR (78.00; IQR: 64.00–80.50%). Samples treated with 100 µM H_2_O_2_ (45.00; IQR: 36.25–54.25%) showed a significantly decrease in MMP than those treated with 100 µM CGA (*p* < 0.01), both 100 µM H_2_O_2_ and 100 µM CGA (68.0; IQR: 57.75–77.50%, *p* < 0.05) and CTR (*p* < 0.01). Moreover, sperm MMP was significantly higher in samples incubated with 100 µM CGA than those treated with both 100 µM H_2_O_2_ and 100 µM CGA (*p* < 0.05) (Figure 2).

### 3.4. Effect of Chlorogenic Acid on Induced LPO in Sperm Samples: MDA Evaluation

**Figure 3:**
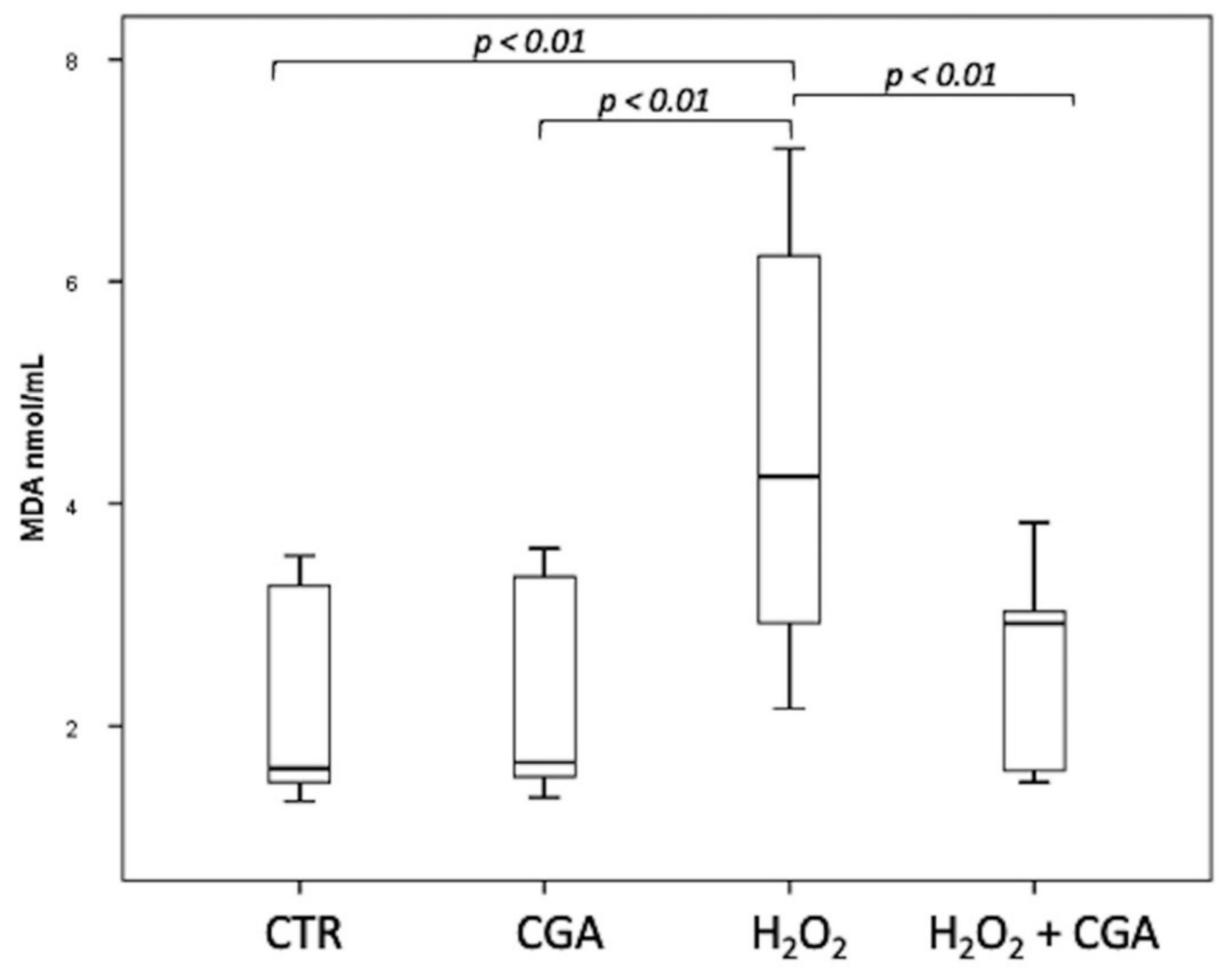
Median (IQR) of MDA levels (nmol/mL) measured in the swim-up media without cells after the different treatments: control (CTR), 100 µM chlorogenic acid (CGA), 100 µM H_2_O_2_ (H_2_O_2_)_,_ 100 µM H_2_O_2_ + 100 µM CGA (H_2_O_2_ + CGA). The experiment was repeated 10 times.

Swim-up selected sperm were treated with 100 µM H_2_O_2_ to induce LPO, with both 100 µM H_2_O_2_ and 100 μM CGA in order to test the potential scavenging activity of this compound and with 100 μM CGA alone. MDA levels were measured, after the different treatments, in the swim-up media without cells, and the results are shown in Figure 3. The levels of MDA significantly increased after H_2_O_2_ treatment (4.24; IQR: 2.89–6.40 nmol/mL) respect to CTR (1.62; IQR: 1.49–3.30 nmol/mL, *p* < 0.01) and CGA alone (1.67; IQR: 1.54–3.38 nmol/mL, *p* < 0.01). The MDA levels in samples incubated with both 100 µM H_2_O_2_ and 100 μM CGA (2.92; IQR: 1.57–3.22 nmol/mL) were significantly lower than those measured in the sample treated with H_2_O_2_ alone (*p* < 0.01) and were not significantly different from those of the CTR (*p* = 0.928) and CGA alone (*p* = 0.957). The MDA values of the samples treated with CGA alone did not show any difference with the CTR (*p* = 1.000).

### 3.5. F_2_-Isoprostane Determination

F_2_-IsoP levels were measured in the media of upper swim-up fraction after treatment with 100 μM H_2_O_2_, with both 100 μM CGA and 100 μM H_2_O_2_, with 100 μM CGA alone and in untreated ones.

F_2_-IsoP levels were significantly higher in samples treated with H_2_O_2_ (322.95; IQR: 275.2–353.875 pg/mL) respect to CTR (2.45; IQR: 1.025–4.425 pg/mL; *p* < 0.001).

The level of F_2_-IsoPs in samples incubated with 100 µM H_2_O_2_ plus 100 μM CGA were significantly lower (42.5; IQR: 30.35–56.725 pg/mL) than those measured in the sample treated with H_2_O_2_ (*p* < 0.01) but higher compared to CTR (*p* < 0.01) and CGA (2.3; IQR: 1.400–3.525 pg/mL, *p* < 0.01). The F_2_-IsoP levels of the samples treated with CGA alone did not show any difference with CTR (*p* = 0.981).

### 3.6. Effect of Chlorogenic Acid on Induced LPO in Sperm Samples: Ultramorphological Evaluation

**Figure 4:**
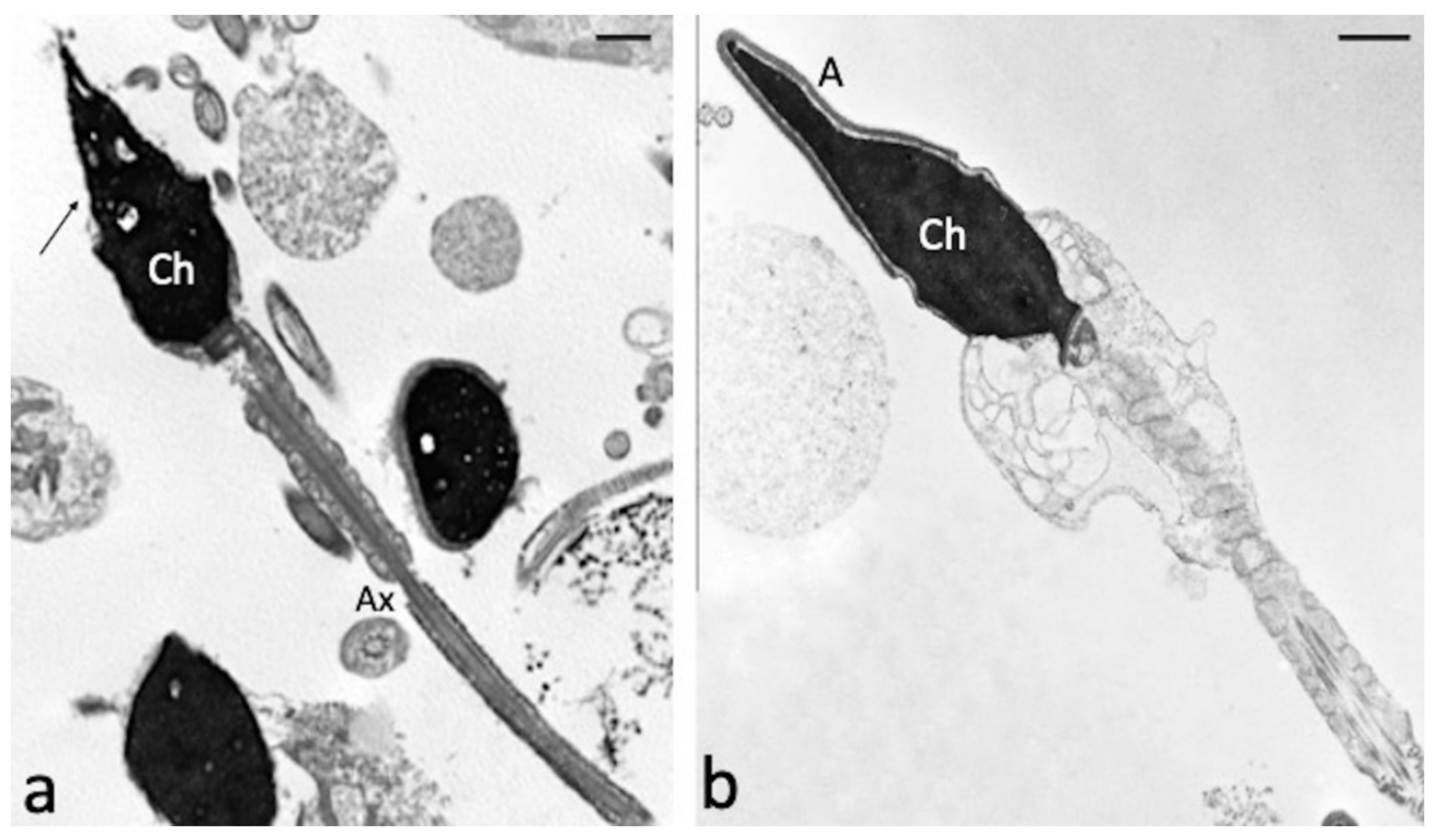
TEM micrographs of swim-up selected spermatozoa treated with 100 µM H_2_O_2_ (**a**) and with both 100 µM H_2_O_2_ and 100 µM CGA (**b**). In figure (**a**), longitudinal and cross sections of spermatozoa with reacted acrosomes (arrow) and broken plasma membranes are observed. The chromatin (Ch) texture appears morphologically normal; axoneme (Ax). When the CGA is present (**b**), the sperm ultrastructure is preserved, the acrosome (A) and the plasma membrane are intact, the chromatin (Ch) is regularly condensed. Bars: 1 μM.

Ultrastructural characteristics of swim-up selected sperm treated with H_2_O_2_ and both 100 µM H_2_O_2_ and 100 µM CGA were analysed by TEM. CGA showed a protective effect against the alterations detected in the samples treated with H_2_O_2_ alone (Figure 4). The percentages of sperm with broken plasma membrane, with reacted or absent acrosome and with alterations in axonemal structure are significantly higher in samples treated with H_2_O_2_ respect to those observed in samples treated with both H_2_O_2_ and CGA (*p* < 0.01, Table 2). The percentage of alterations of chromatin texture was similar in both samples.

### 3.7. Cryopreservation Experiments

**Figure 5:**
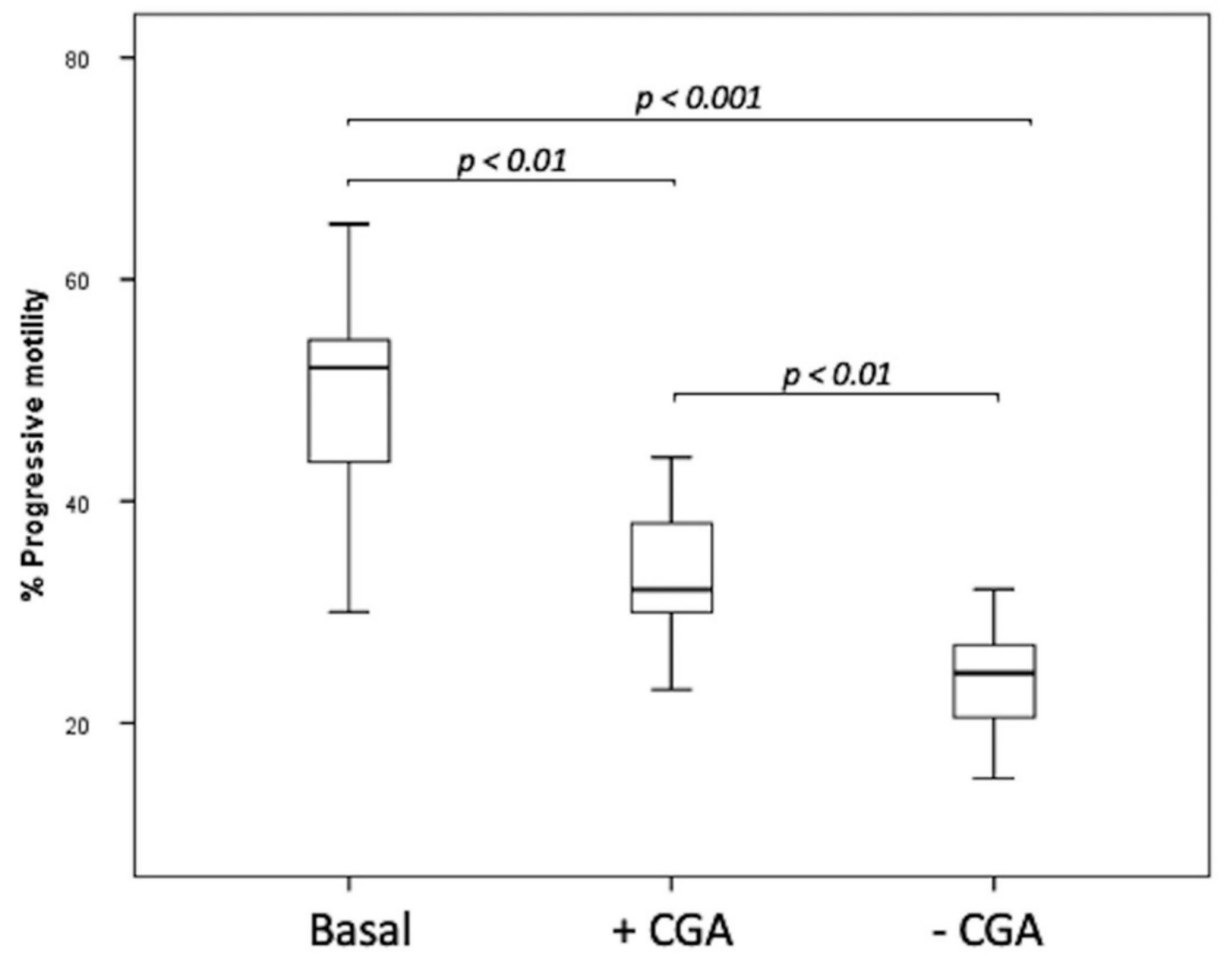
Median (IQR) of progressive sperm motility percentage evaluated in basal samples and frozen samples supplemented with (+CGA) and without CGA (−CGA). The experiments were performed on 8 samples and assessed twice by 2 different examiners.

The step 2 of this research concerns cryopreservation experiments performed in the semen of 8 patients. After thawing, the percentage of progressive sperm motility in basal sample (52.5, IQR: 46–55.75%) before freezing was higher than that observed in both frozen untreated (24.5, IQR: 20.25–27.00%, *p* < 0.001) and CGA treated specimens (32.5, IQR: 30.00–38.75%, *p* < 0.01) (Figure 5). The percentage of progressive motility was higher in frozen samples supplemented with CGA than that observed in the untreated one (*p* < 0.01) (Figure 5).

The MDA levels, DNA integrity and high MMP percentages assessed in frozen-thawed, treated and untreated, samples are reported in Table 3.

MDA levels were significantly higher in untreated samples than those observed in samples supplemented with 100 µM CGA (*p* < 0.01). The percentage of the sperm with dsDNA significantly increased in frozen samples supplemented with 100 µM CGA compared with frozen untreated samples (*p* < 0.01). The percentage of spermatozoa with high MMP in CGA supplemented samples was significantly increased respect to that observed in the untreated frozen specimens (*p* < 0.01).

### 3.8. Immunocytochemistry

**Figure 6:**
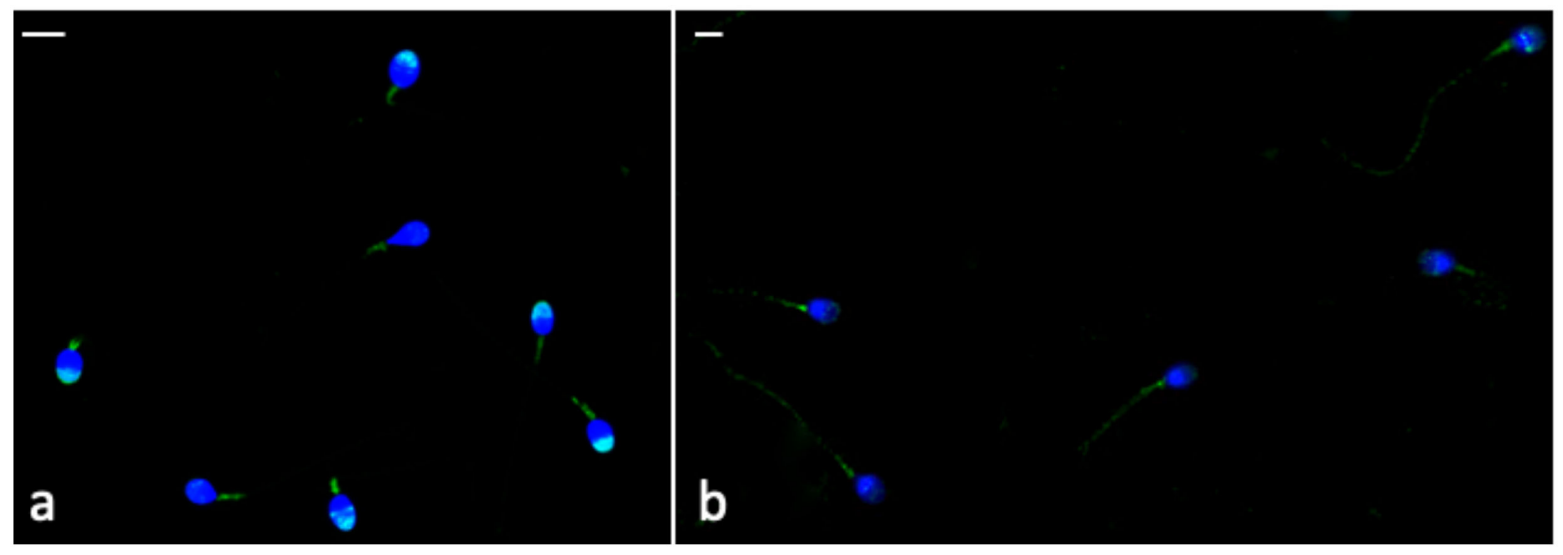
UV micrographs of frozen-thawed sperm treated with 100 µM CGA (**a**) and untreated (**b**). In (**a**), the labelling in the midpiece and acrosome of most treated frozen-thawed spermatozoa is shown. In (**b**), the untreated frozen-thawed sperm show a labelling in the midpiece and along the tail. Bars: 6 μM.

The Phospho-AMPKα labelling was brighter in the frozen-thawed sperm treated with CGA (Figure 6a) than that observed in untreated samples (Figure 6b). In frozen–thawed samples (treated and untreated) the labelling was present in the midpiece; however, in CGA-treated samples, the percentage of sperm with the labelling in the head, mainly in the acrosome (Figure 6a), was higher than that detected in untreated samples (Table 4, *p* < 0.001).

The percentage of sperm with labelling all along the tail (Figure 6b) was higher in untreated frozen-thawed samples than the treated ones (*p* < 0.001).

## 4. Discussion

The in vitro experimental protocol used in this research pointed out that CGA is worthy of investigation for its interesting antioxidant and protective properties in the field of human sperm handling.

After the demonstration that this compound did not interfere with sperm motility, we decided to explore the ability of CGA to prevent OS induced in vitro by H_2_O_2_ on swim-up-selected human sperm population as homogeneous as possible.

The choice of concentration for the experiments was supported by a recent observation in which the supplementation of 100 μM CGA played a beneficial effect on the quality of frozen-thawed boar spermatozoa [30]. In addition, our data indicated a positive effect of this concentration on sperm motility. CGA is involved in the regulation of the components of the purinergic system [39] that has been accepted as an important modulator of physiological phenomena including the regulation of several functions in the male and female reproductive tract organs [40]. For this reason, a direct effect of CGA on P2X receptors expressed by sperm cells cannot be excluded.

Finally, based on results acquired during the in vitro studies, we used CGA to supplement media used for sperm cryopreservation.

The study of natural compounds endowed with antioxidant properties is appealing, both for male infertility treatment and the development of new strategies for media supplementation used for semen handling and during cryopreservation protocols. Many natural compounds with scavenging activity against LPO induced in vitro in human spermatozoa were studied [18,19,41,42]. Recently, Rabelo et al. [32] observed that the addition of CGA to the semen extender improved the quality of boar sperm processed by Percoll technique or unprocessed, better than that obtained with vitamin E supplementation. The benefits expected from the use of CGA in human semen were observed in this research.

The incubation with 100 μM CGA alone did not trigger oxidative damage. This result should not be underestimated by considering the possible pro-oxidant effect of antioxidants if used in high concentration or in combination [43].

The anti-ROS effect of CGA, detected by its protective activity against LPO induced by in vitro H_2_O_2_ treatment, was suggested by measuring both MDA and F_2_-IsoPs. MDA represents one of the decay end-products of LPO, probably the most used in the assessment of oxidative insult, however other compounds such as 4-hydroxynonenal and isoprostanes can be measured as indicators of OS [10,11]. To confirm the MDA results, F_2_-IsoPs, products of arachidonic acid oxidation, were also assessed. Our results showed that H_2_O_2_ treatment of swim-up selected sperm leaded to the formation and release of F_2_-IsoPs in the medium and the addition of CGA almost totally (over 80%) reduced this oxidative event. Thus, detailed data on fatty acid stability in presence of CGA may be relevant for further investigations on CGA antioxidant properties.

A protective effect of CGA on DNA integrity as well as on MMP was observed. These results are supported by the observation that CGA protected in vitro porcine oocytes from the negative effects of heat stress and from DNA damage [44]. In addition, since mitochondria generate energy to support motility, high percentage of sperm with high MMP represents an index of cellular activity, vitality, and integrity of mitochondrial cristae.

To study the protective activity of CGA from a morphological point of view, a deep analysis of the different sperm organelles was performed using TEM, a useful method to explore the ultrastructure of human sperm treated in vitro with different compounds [18,19]. TEM analysis confirmed the protective effect of CGA, which was able to preserve membranes and acrosomes, structures both particularly affected by H_2_O_2_ treatment. The sperm chromatin was not particularly altered by the mild oxidative stress treatment because its tight packaging due to the replacement of histones by protamines [45].

After the in vitro demonstration of protective and antioxidant activities of CGA, we applied the acquired knowledge to the field of sperm cryopreservation that allows for the storage of cells for long periods. However, the use of extremely low temperatures makes cells susceptible to oxidative insults during freezing and thawing processes. The main cryogenic injuries regard sperm motility, viability, membrane integrity, DNA fragmentation and mitochondrial activity [46]. Sperm cryopreservation was reported to damage even crucial genes involved in fertilization and early embryo development [7] and to increase DNA fragmentation caused by high OS levels rather than to caspase activation [47]. The supplementation of semen extenders with antioxidants leaded to an increase in sperm quality after thawing in several species [48,49,50], including humans [51,52,53]. Recently, Sun et al. [50] showed that the addition of resveratrol to extender improved sperm motility, membrane, and acrosome integrity, MMP and protected boar sperm against OS during semen preservation and fast cooling process.

In the present study, the supplementation of cryopreservation medium with CGA displayed a protective effect on sperm motility after thawing, although both frozen samples showed a decrease in progressive sperm motility compared to basal specimen before freezing. It is reported that the percentage of motile spermatozoa can decrease from 50.6% to 30.3% after cryopreservation [54], and this phenomenon is true, in particular, for low quality semen.

The benefits of CGA treatments on LPO, MMP and DNA integrity were also evident. The observed strong protective action on DNA is extremely important considering that spermatozoa are transcriptionally silent and have no repair mechanisms, making them more susceptible to attack of ROS highly produced during cryopreservation.

Pereira et al. [31] suggested that CGA has a potent protective action and antioxidant activity in liquid storage conditions of boar semen, even more stable than that displayed by vitamin E. This stability in long period storage is particularly attractive for the use in cryopreservation protocols.

Namula et al. [30] has also reported a protective effect of CGA during boar sperm freezing; however, the present study is the first report of the effect of CGA on human sperm in cryopreservation conditions. The cryotolerance and cold sensitivity of spermatozoa are mainly due to the lipid composition of the sperm plasma membrane. Omega-3/omega-6 ratio, fatty acid profile, as well as size and shape are different in spermatozoa of the different species and these parameters can affect the reaction to cryopreservation [46].

Finally, another aspect considered in study accounts for the immunolocalization of Phospho-AMPKα in frozen-thawed sperm both treated with CGA and untreated. Recently, Naveed et al. [29] revised the biological and pharmacological role of CGA reporting that this compound was able to increase the AMPK phosphorylation.

Our immunolocalization results in human sperm agree with those obtained by Hurtado de Llera et al. [55] that showed the Phospho-AMPKα labelling at the acrosome, equatorial segment and midpiece levels of boar spermatozoa. Spermatozoa treated with CGA showed a brighter labelling than untreated samples suggesting that this compound might play a role in increasing AMPK phosphorylation.

In frozen-thawed sperm treated with CGA, Phospho-AMPKα was localized in the acrosome and midpiece suggesting a protective action of CGA at the membrane level, structure usually damaged in the freezing process, as previously confirmed by MDA. Furthermore, in the untreated samples the weak labelling all along the tail and in the midpiece suggested a decrease in ATP due to low-temperature stress, considering that AMPK is a cellular sensor of energy status and is inactive in presence of low ATP levels [34,56]. These results, although preliminary, suggest that the protective action of CGA on spermatozoa in cryopreservation protocols may be mediated by an AMPK activation mechanism, indicating the importance of this kinase in human sperm function. As stated before, we can hypothesize that CGA may act via purinergic system [39] since it is reported that this system can regulate the activation of AMPK [57]; obviously, these hypotheses must be confirmed by dedicated studies specifically in the male reproductive tract.

Several studies reported that AMPK activation improved the sperm quality by regulating the energy metabolism and the fertilization ability of cryopreserved sperm [58,59]. Recently, Feng et al. [60] showed that rosmarinic acid supplementation during boar semen storage at 17 °C was able to activate AMPK phosphorylation, resulting in increased sperm quality, antioxidant ability and sperm-zona pellucida binding capacity.

## 5. Conclusions

CGA is not toxic for sperm motility, DNA integrity and MMP and has protective activity both against OS induced with H_2_O_2_ and during the cryopreservation procedure where OS is exacerbated. This compound deserves other studies, since it could be used to supplement culture media during gamete manipulation and protocols related to ART procedures.

## Figures and Tables

**Table 1:** Median (IQR) of progressive sperm motility percentage evaluated in control (CTR) and samples treated with 50 µM, 100 µM, 200 µM and 500 µM chlorogenic acid (CGA). The experiment was repeated 10 times.

Parameter	CTR	CGA 50 µM	CGA 100 µM	CGA 200 µM	CGA 500 µM
Progressive sperm motility %	53.5 (49.5–65.0)	55.0 (51.50–65.75)	72.0 * (59.0–79.25)	67.5 (56.25–77.75)	59.5 (55.5–71.0)

* CTR vs. CGA (100 µM): *p* < 0.05.

**Table 2:** Median (IQR) of percentages of altered sperm structures analysed by TEM in swim-up selected sperm treated with 100 µM H_2_O_2_ and 100 µM H_2_O_2_ + 100 µM CGA. The experiments were repeated 3 times and assessed twice by different examiners.

% Altered Sperm Structures	H_2_O_2_	H_2_O_2_ + CGA
Altered acrosome	87.0 (76.75–92.50)	29.0 (25.75–33.75) *
Altered chromatin	44.5 (42.50–54.00)	41.5 (39.50–45.25)
Altered axoneme	50.0 (48.25–52.00)	33.5 (30.00–45.75) *
Broken plasma membrane	84.0 (79.50–87.25)	30.0 (21.50–32.75) *

H_2_O_2_ vs. H_2_O_2_ + CGA * *p* < 0.01.

**Table 3:** Median (IQR) of MDA levels, MMP and dsDNA percentages evaluated in frozen samples: supplemented with CGA (+CGA) and untreated (−CGA). The experiments were performed on 8 samples and assessed twice by 2 different examiners.

Frozen Samples	MDA Levels nmol/mL	Sperm with High MMP %	Sperm with dsDNA %
+CGA	1.93 * (1.063–2.613)	32.5 * (30.00–38.75)	97.0 * (93.50–98.75)
−CGA	3.238 (2.755–4.878)	24.5 * (20.25–27.00)	60.5 * (57.25–63.00)

+CGA vs. −CGA * *p* < 0.01.

**Table 4:** Median (IQR) of percentages of sperm head and tail labelled with Phospho-AMPKα antibody in frozen-thawed samples: supplemented with CGA (+CGA) and untreated (−CGA). The experiments were performed on 8 samples and assessed twice by two different examiners.

Phospho-AMPKα Labelling	*%* Sperm Head	*%* Sperm Tail
+CGA	56.00 * (49.50–60.50)	16.00 * (12.75–20.50)
−CGA	26.00 (23.50–28.25)	55.50 (49.50–61.25)

+CGA vs. −CGA * *p* < 0.001.

## Data Availability

Data is contained within the article and are available on request from the corresponding author.
